# Association between aflatoxin B1 occupational airway exposure and risk of hepatocellular carcinoma: a case-control study

**DOI:** 10.1007/s13277-014-2231-3

**Published:** 2014-06-25

**Authors:** Hao Lai, Xianwei Mo, Yang Yang, Ke He, Jun Xiao, Chao Liu, Jiansi Chen, Yuan Lin

**Affiliations:** 1 Department of Gastrointestinal Surgery, Affiliated Cancer Hospital of Guangxi Medical University, 71 Hedi Road, Nanning, 530021 Guangxi Autonomous Region China; 2 Department of Head and Neck Surgery, Affiliated Cancer Hospital of Guangxi Medical University, Nanning, 530021 Guangxi Autonomous Region China

**Keywords:** Hepatocellular carcinoma, Aflatoxin B1, Airway exposure, Case-control study

## Abstract

The aim of this study was to determine the airway exposure of sugar and papermaking factory workers to aflatoxin B1 (AFB1) and to explore the potential association between AFB1 airway exposure and the risk of hepatocellular carcinoma (HCC) in a case-control study. Dust samples were collected from the sugarcane bagasse warehouse, and presser and paper production workshops. Blood samples were collected from 181 workshop employees and 203 controls who worked outside the workshop. AFB1 albumin adducts were detected using a double antibody sandwich enzyme-linked immunosorbent assay (ELISA). To explore the association between AFB1 airway exposure and the risk of HCC, the medical records of 68 HCC patients who worked in a sugar and papermaking factory between January 1994 and December 2013 were analyzed. A questionnaire was used to collect information from 150 healthy controls who worked for the same company and lived near the factory. AFB1 was detected in the dust samples, but could not be detected in any of the rice samples. An analysis of serum samples revealed serum AFB1 albumin adducts in 102 (56.35 %) of the study participants. However, in the control group, only 12 (5.9 %) individuals had detectable levels of AFB1 albumin adducts. Those with airway exposure to *Aspergillus flavus*-contaminated dust had an elevated risk of HCC compared to those without exposure (odds ratio, 5.24; 95 % confidence interval, 2.77–9.88; *P* = 0.00). The findings of this study indicate that occupational AFB1 airway exposure might be associated with the risk of AFB1-related HCC among the population that was used in this study. Intervention programs aimed at reducing exposure to inhalational AFB1 are needed urgently. Additional suitably designed, multicenter, prospective studies using large samples are needed to further confirm the results.

## Introduction

Hepatocellular carcinoma (HCC) is the fifth most common human cancer, with approximately 750,000 new cases occurring worldwide each year [1]. Eighty percent of global HCCs occur in developing countries [2], including the People’s Republic of China [3]. Chronic hepatitis B virus (HBV) infection and exposure to aflatoxin B1 (AFB1) are the principal causes of HCC in China and in other regions of the world [4].

Aflatoxins (AFB1, AFB2, AFG1, and AFG2) are known to be carcinogenic to both humans and animals, with AFB1 being the most potent hepatotoxic and hepatocarcinogenic agent [4]. The principal source of AFB1 exposure is consumption of various food products, particularly cereals, oleaginous seeds, cocoa, coffee, grapevine, wine, fruits, spices, and dried fruit [5]. Numerous studies have demonstrated that a linear correlation exists between serum AFB1 dietary exposure and the risk of HCC [6–10]. In recent years, primary prevention strategies against AFB1 dietary exposure and the HBV, which are the other two principal etiological risk factors in HCC, have offered hope of lowering HCC rates in China [11]. Examples of strategies include HBV vaccination of infants and food safety procedures to control AFB1 contamination [11]. However, the incidence of HCC remains high [2]. According to our clinical observations, the prevalence is particularly elevated in specific populations, such as papermill and sugar factory workers. The dietary habits and HBV infective rate of these workers are similar to those of the rest of the population. Thus, we considered whether another AFB1 exposure route might contribute to HCC in these workers.

Inhalation of contaminated airborne dust is an additional route of exposure that researchers have largely ignored. Airborne aflatoxin was previously detected in dust samples during the handling of contaminated corn [12], and the agricultural workers were directly exposed to the airborne aflatoxin in grain dust through inhalation [5].

Numerous studies that include a focus on China and other countries have established a strong association between AFB1 dietary exposure and the risk of HCC [13–16]. However, only a small number of studies have included an examination of airway exposure to AFB1 [5, 17–19], and the role that inhaled AFB1 plays as an occupational risk factor in the development of HCC is largely unknown. The aim of this study was to determine the AFB1 airway exposure in sugar and papermaking factory workers and to explore the association between AFB1 airway exposure and HCC in this population by employing a case-control study.

## Materials and methods

**Fig. 1:**
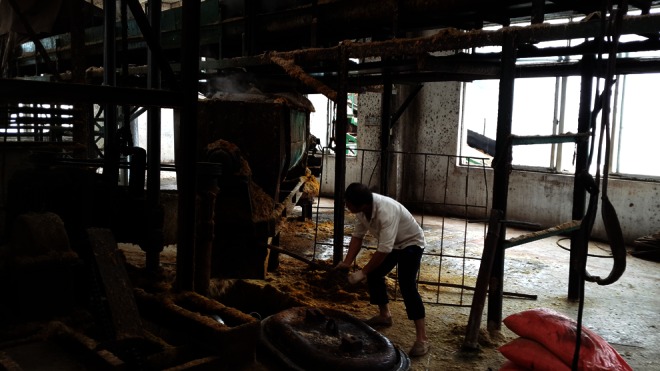
A sugar factory worker who did not use respiratory protection or other type of protective device at work

The Research Ethics Committee of the Affiliated Cancer Hospital of Guangxi Medical University approved this study. This study was conducted between October 2013 and March 2014 in a sugar and papermaking factory located in the Guangxi Autonomous Region of China. It is the largest sugar factory in Guangxi. The principal operations conducted in this factory are sugar production and papermaking using sugarcane, which a previous study demonstrated is a potential source of fungal growth [20]. The workers’ airways were directly exposed to the AFB1-contaminated dust when producing sugar and paper. They did not always use respiratory protection or other types of protective devices at work (Fig. 1).

### Part I: determination of AFB1 airway exposure

#### Dust sample collection

**Fig. 2:**
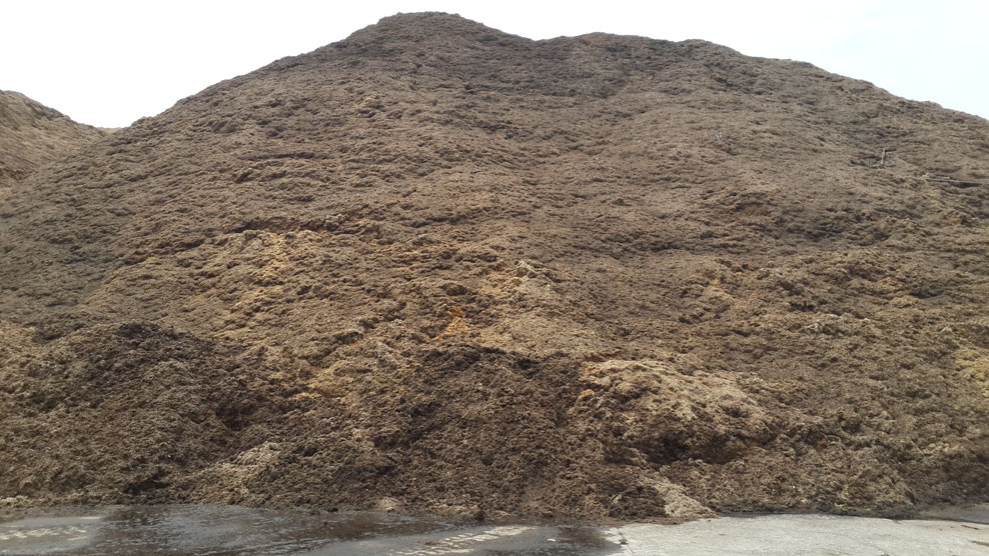
Dust sample in sugarcane bagasse warehouse

**Fig. 3:**
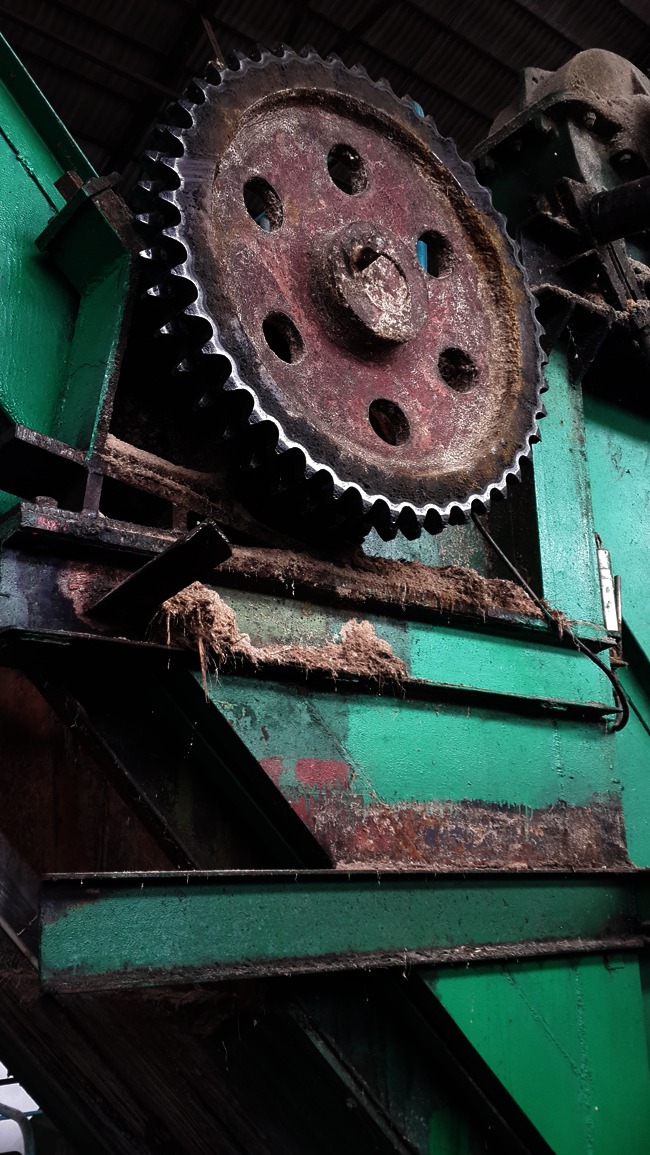
Dust sample in the presser workshop

Dust samples were collected from the sugarcane bagasse warehouse (Fig. 2) and presser (Fig. 3) and paper production workshops of the mentioned factory because these were regarded as the most likely sites of contamination. AFB1 was isolated from the dust samples using the method described by Autrup et al. [17]. Dust samples (0.9 mg) were suspended in 5 ml of 80 % methanol, and 0.1 mg of sodium chloride was added to the turbid liquid. After 30 min, these extracts were centrifuged for 20 min, and the supernatants were collected. This procedure was repeated three times, and all the supernatants were pooled. The extract (3 ml) was adjusted using 10 % methanol, and 10 ml of this solution were loaded into aflatoxin test affinity column. After washing with distilled water, the AFB1 were eluted using 100 % methanol.

#### Detection of AFB1 in dust samples from the sugar and papermaking factory

The concentration of AFB1 in the dust samples was analyzed using an indirect competitive enzyme linked immunosorbent assay (ELISA) (Reagen, USA). ELISA wells were coated using 100 ng/ml of AFB1 of bovine serum albumin (BSA) in a sodium carbonate buffer with pH 9.6 (150 ml/well) and left overnight at 4 °C. A phosphate-buffered saline was used to wash the ELISA plates, and BSA was added to the plates and allowed to stand at 37 °C for 1 h. Phosphate-buffered saline containing 0.05 % Tween (PBST) was used to wash the ELISA plates again, and 100 ml of AFB1 standards ranging from 25 ng/ml to 10 pg/ml was then added to the plates. Preincubation was conducted using 50 ml of antiserum diluted in PBST–BSA (1:6,000), and maintained for 45 min at 37 °C. The extraction solution from the dust samples and aqueous methanol-KCl were added to wells at 1:10 dilution in PBST–BSA. Goat anti-rabbit immunoglobulins conjugated to alkaline phosphatase were used at a 1:4,000 dilution to detect rabbit antibodies that were attached to AFB1–BSA. *S*-Nitrophenyl phosphate (0.5 mg/ml) was used as a substrate. Absorbance was recorded at 450 nm using an ELISA plate reader (Biorad-680, USA) after incubation at 28 °C for 45 min to 1 h in the dark. Standard curves were obtained by plotting log_10_ values of AFB1 dilutions at A450. The AFB1 (ng/ml) in the samples was determined by referring to standard curves: AFB1 mg/kg of dust sample = aflatoxin (ng/ml) in sample × buffer (ml) × extraction solvent (ml)/sample weight (g). To test the recovery of AFB1, 20 g of dust was mixed using pure AFB1 (Sigma, St. Louis, MO, USA) to produce concentrations ranging from 5 to 100 mg/kg.

#### Dietary AFB1 analysis

Fifty rice samples were collected randomly from the participants and from the kitchen in the factory. AFB1 was analyzed through an indirect competitive ELISA using the process that was described earlier.

#### Selection of the study subjects and blood sample collection

Employees were enrolled in the study group if they met the following criteria: (1) they have worked in the sugarcane bagasse warehouse and presser or paper production workshops for at least 4 weeks; (2) they had not eaten any moldy foods within 3 months; and (3) they had not been working for any other agribusiness in the 3 months prior to the start of this study. We excluded participants from the study group if they met any of the following criteria: (1) they did not work in the mentioned workshops; (2) they had eaten moldy food once within 3 months; and (3) they had worked in any other agribusiness in the 3 months prior to the start of this study.

We selected participants for the control group if they met the following criteria: (1) they worked for the sugar mill or papermaking factory and in positions outside of it, such as administrative work in an office or as workers in a store nearby; (2) they had not eaten any moldy foods within 3 months; and (3) they had not been working in any other agribusiness in the 3 months prior to the start of this study. We excluded participants if they met any of the following criteria: (1) they had directly inhaled the dust while working for the mentioned company once within 3 months; (2) they had eaten moldy food once within 3 months; and (3) they had worked in any other agribusiness activity in the 3 months prior to the start of this study.

At least 181 workshop employees and 203 controls were enrolled in this study. Blood samples (5 ml) were collected from these study participants, and serum was isolated from the blood samples by centrifugation and stored at –20 °C until further analysis.

#### Determination of serum AFB1 albumin adducts level

A double antibody sandwich ELISA for humans was used to detect serum AFB1 albumin adducts. The sensitivity of the analysis using ELISA was 6 pg/mg albumin (Yisen, Shanghai, China). Serum samples were thawed for 1 h before conducting the ELISA analysis. Standards were diluted according to the manufacturer’s instruction. A total of 10 μl of the test sample was diluted using 40 μl sample diluent. A reagent was not added to the blank control. After closing the plate using a closure plate membrane, the plates were incubated at 37 °C for 30 min. The closure plate membrane was then opened and the liquid in each well was discarded and swung, taking care not to leave any liquids in each well. A wash buffer was added to the wells. After 30 s, the plate was inverted, and residual liquid was tapped out. This step was repeated five times. Subsequently, 50 μl of EIA kit was added to the test well again. The plates were incubated and washed again, as previously described.

In the next step, 50 μl of chromogenic agent A and 50 μl of chromogenic agent B were added to each well in turn, and the plate was kept in the dark for 30 min at 37 °C. Subsequently, 50 μl of stopping solution was added to the wells, thus changing the color of the liquid to yellow. The absorption was read at 450 nm.

### Part II: assessment of AFB1 airway exposure and risk of HCC

#### Study population

To evaluate AFB1 inhalation and the risk of HCC, a case-control study was conducted, which comprised 68 HCC patients who worked at the sugar mill and papermaking factory between January 1994 and December 2013, and 150 healthy controls who worked for the same company during the same period. The records of the HCC patients were obtained from community and governmental hospitals, whereas the control group participants were selected from the mentioned company using a random sampling method. The study participants were excluded from the control group if they met any of the following criteria: (1) they had been working for their company for less than 5 years; and (2) they did not reside at the same residence. A questionnaire was used to collect information on the gender, HBV infection status, alcohol intake, smoking status, moldy food intake, and occupational airway exposure to dust of the controls. We were unable to compare the serum AFB1 albumin adduct levels of the cases and controls because the information on the HCC patients was obtained by conducting a retrospective analysis, most of the patients had died, and serum samples that had been collected when they were alive were impossible to obtain.

### Statistical analysis

In Part I, an independent samples *t* test was performed to evaluate whether the serum AFB1 albumin adduct levels of the workshop workers and controls were statistically significantly different. A one-way analysis of variance was performed to evaluate whether the concentration of AFB1 in dust samples, which were collected from the sugarcane bagasse warehouse and the presser and paper production workshops were significantly different. A chi-square test was also used to assess whether the characteristics of the participants in the two groups were different. In Part II, Fisher’s exact test or a chi-square test, whichever was appropriate, were used to examine the differences between the cases and the controls. The odds ratio (OR) and 95 % confidence intervals (95 % CI) for HCC risk were calculated using an unconditional logistic regression, and a full assessment of potential confounding factors was conducted. Statistical analysis was performed using the Statistical Package for the Social Science, version 16.0 (SPSS 16.0), and a *P* value <0.05 was considered statistically significant.

## Results

Fifteen dust samples were collected from the workshop (sugarcane bagasse warehouse, and the presser and paper production workshops). The concentration of AFB1 in dust samples, which were collected from the sugarcane bagasse warehouse and the presser and paper production workshops, were 7.2 ± 1.30, 8.0 ± 1.23 and 8.6 ± 1.82 μg kg^−1^, respectively (Table 1). The concentration of AFB1 in the dust samples was not statistically significant in these sites (*P* = 0.35). AFB1 was not detected in any of the rice samples.

**Table 1:** Concentration of AFB1 in dust samples from different workshop

Workshop	Concentration of AFB1 in dust samples
Sugarcane bagasse warehouse	7.2 ± 1.30 μg kg^−1^
Presser workshop	8.0 ± 1.23 μg kg^−1^
Paper production workshop	8.6 ± 1.82 μg kg^−1^
*P* value	0.35

The demographic data are listed in Table 2. A statistically significant difference was not detected for the age, gender, body mass index (BMI), daily work periods, HBV infection, and moldy food intake of the two groups. However, the difference in the serum AFB1 albumin adduct levels of the workshop workers and the controls was statistically significant. Serum AFB1 albumin adducts were detected in 102 (56.35 %) workshop employees, with values ranging from 8 to 212 pg/mg albumin (mean value 38.51 ± 44.80 pg/mg albumin). In contrast, only 12 (5.9 %) controls had detectable levels of AFB1 albumin adducts, with values ranging from 8 to 26 pg/mg albumin (mean value 15.58 ± 6.42 pg/mg albumin).

**Table 2:** Characteristics of the involved participators

Information	Workshop workers (*n* = 181)	Controls (*n* = 203)	*P* value
Age (years)	40.38 ± 9.32	38.13 ± 9.98	0.12
Gender (male/female)	127/54	139/64	0.72
BMI	22.22 ± 2.84	22.56 ± 2.84	0.64
Daily work periods (h)	7.89 ± 0.78	7.85 ± 0.56	0.63
HBV infection			
Positive	16	22	0.51
Negative	165	181	
Moldy food intake			
Yes			
No			
Serum AFB1 albumin adducts	38.51 ± 44.80 pg/mg albumin	15.58 ± 6.42 pg/mg albumin	0.00
Positive	102	12	0.00
Negative	79	191	

Table 3 shows the results of a comparative analysis of the risk of HCC with the principal etiological factors in the cases and controls. We found that the risk of HCC was associated with exposure to airborne dust (OR, 5.24; 95 % CI, 2.77–9.88; *P* = 0.00) and HBV infection (OR, 4.46; 95 % CI, 2.02–9.85; *P* = 0.00). However, no statistically significant risk was observed with respect to gender (OR, 1.20; 95 % CI, 0.67–2.16; *P* = 0.54), alcohol intake (OR, 0.88; 95 % CI, 0.50–1.56; *P* = 0.66), smoking status (OR, 0.87; 95 % CI, 0.49–1.56; *P* = 0.64), and family history of HCC (OR, 1.72; 95 % CI, 0.57–5.16; *P* = 0.33).

**Table 3:** Demographic characteristics of HCC cases and controls

Variables	HCC cases (*n* = 68)	Controls (*n* = 150)	OR (95 % CI)	*P* value
Gender				
Male	42	86	1.20 (0.67–2.16)	0.54
Female	26	64		
Alcohol intake				
Yes	35	82	0.88 (0.50–1.56)	0.66
No	33	68		
Smoking status				
Yes	39	91	0.87 (0.49–1.56)	0.64
No	29	59		
HBV infection				
Positive	19	12	4.46 (2.02–9.85)	0.00
Negative	49	138		
Family history of HCC				
Yes	6	8	1.72 (0.57–5.16)	0.33
No	62	142		
Exposure to airborne dust				
Yes	50	52	5.24(2.77–9.88)	0.00
No	18	98		

*HBV* hepatic B vivus, *HCC* hepatocellular carcinoma, *OR* odds ratio

## Discussion

HCC is one of the principal cancer types that affects the people living in the Guangxi Zhuang Autonomous Region, where most disease occurs due to environmental exposure to AFB1 [8–10]. AFB1 exposure occurs predominantly through the food chain, but inhalation is an additional route of exposure. Airborne exposure to AFB1 principally occurs in workers who are in contact with *Aspergillus flavus*-contaminated dust [5, 17, 21]. The strong association between AFB1 dietary exposure and the risk of HCC has been established [4]. However, the association between airway exposure to AFB1 and the risk of HCC is largely unknown, and studies in this field are apparently nonexistent. The present study showed that AFB1 airway exposure might result in positive serum AFB1 adducts and be associated with a risk of HCC.

Numerous studies have revealed that poultry production and rice mill workers who are indirect contact with grain dust are often exposed to AFB1 [5, 17, 18, 21, 22]. This study demonstrated that sugar refinery workers are also at risk of inhalational exposure to AFB1. The sugarcane plant (*Saccharum officinarum*) is the predominant crop used in sugar production in the province of Guangxi. The sugar present in the stem of *S. officinarum* is the principal source of fungal growth [20]. The sugar factory workers in this study might constitute a high-risk population due to the absence of precautions against *A. flavus* in this factory. Based on a conservative estimation, the temperature in the factory workshop was 35–40 °C. Due to the factory’s high temperature, the workers were reluctant to use respiratory protection or other types of protective devices. They also seemed to have no knowledge about the need for protection.

Pulmonary exposure to AFB1-laden dust at work is common, and most studies of AFB1 airway exposure have focused on its carcinogenic effect on the lung and its related mechanisms [23–26]. The carcinogenic effect of AFB1 airway exposure on other organs has not been studied. The detection of serum AFB1 albumin adducts in the current study suggests that the liver might be a potential target for airway exposure to AFB1. In the study, 102 of the 181 workshop employees (56.35 %) who were directly exposed to *A. flavus*-contaminated dust had detectable levels of serum AFB1, with values ranging from 8 to 212 pg/ml. This finding is consistent with the results of previous studies [5, 17], which reported positive serum AFB1 albumin adducts among workshop workers who had been directly exposed to grain dust. In the current study, 12 controls had detectable levels of serum AFB1 albumin adducts. This might be attributed to experimental error. The absence of detectable levels of serum AFB1 albumin adducts in some of the workshop workers might be caused by individual differences or different concentrations of dust in some areas.

Serum AFB1 albumin adducts are of substantial interest because they reflect damage to critical molecular targets and damage to the absorption, distribution, and metabolism of AFB1 biotransformation in vivo [27]. Although AFB1 is harmless, members of the cytochrome P450 family convert the innocuous parent molecule of AFB1 into mutagenic and carcinogenic intermediates. AFB1 is converted into AFB1-8,9-exo-epoxide, which, in turn, is converted into the 8,9-dihydroxy-8-(*N* ^7^)guanyl-9-hydroxy AFB1 adduct [4].

To date, all animal models that have been exposed to AFB1 have developed HCC [4]. Numerous studies found a dose–response relationship between levels of AFB1 adducts, the risk of HCC, and the number of years of exposure toAFB1, as measured by years living in a high AFB1 exposure region [6, 8–10]. The findings of this study are consistent with studies that found that AFB1 airway exposure was associated with positive serum AFB1 adducts, which might be correlated with an increased risk of AFB1-related HCC. As shown in Table 3, both airborne dust exposure and HBV infection contributed to the development of HCC, but the airborne dust exposure played a greater role than HBV infection, which is a leading public health and social problem in China and is associated with the development of HCC [28]. In this study, the difference in levels of detectable AFB1 adducts between the workshop workers and the controls was statistically significant, and the HBV infection rate between them was similar. Thus, we conclude that AFB1 airway exposure was the principal cause of the HCC in this study.

Although the AFB1 DNA adducts have greater relevance in relation to HCC [29], we used AFB1 albumin adducts to assess AFB1 exposure. One of the advantages of AFB1 albumin adducts is that their half-time ranges between 2 and 3 weeks [30]. In contrast, the half-time of AFB1 is nearly 30 min and that of AFB1 DNA adducts in animals is less than 24 h. Additionally, measurements of albumin adducts are particularly useful because they require simple, facile immunoassays that can be applied to large numbers of samples in field studies [31]. A previous animal study reported a substantial correlation between the binding of AFB1 to serum albumin and liver DNA [32]. The same study showed that that binding to serum albumin was a good biomarker of binding to target cell DNA, even at different doses of AFB1 [17].

In summary, the results of the current study suggest that sugar and papermaking factory workers were exposed inhalationally to AFB1 and that this exposure might be associated with the development of HCC in this population. Therefore, intervention programs are needed urgently. First, because the temperature in workshop is elevated and workers are reluctant to use respiratory protection, suitably planned exhaust systems should be installed to decrease the temperature in the workshops. Second, the workers should be advised to wear protective masks when they work. Third, the company should reduce the level of AFB1 in the raw material (*S. officinarum*) because it is the principal source of fungal growth. Fourth, a chemoprevention program involving an intervention that employs green tea should be conducted among workshop workers because numerous studies have indicated that green tea could effectively modulate the AFB1 metabolism and metabolic activation [33], and inhibit the initiation and promotion steps of AFB1 hepatocarcinogenesis [34].

This study has numerous limitations. First, the association between AFB1 airway exposure and the risk of HCC was assessed in a small number of individuals in a single center using retrospective data. Second, the number of HCC cases in each year was small. Third, we could not determine the AFB1 adduct level in the HCC patients because most of them had died. Therefore, suitably designed, multicenter, prospective studies with large samples should be conducted to assess airway exposure to AFB1 and the risk of HCC. Fourth, potential bias might be produced because we used random sampling to determine the AFB1 contamination of the dust samples. Lastly, uncontrolled or unmeasured confounding factors, such as the effects of other environmental factors or gene polymorphisms, which also potentially produce biases, might have affected the incidence of HCC.

## Conclusions

The findings of this study indicate that occupational AFB1 airway exposure might be associated with the risk of AFB1-related HCC among the population that was used in this study. Intervention programs aimed at reducing exposure to inhalational AFB1 are needed urgently. Additional suitably designed, multicenter, prospective studies using large samples are needed to further confirm the results.
